# Potency assays for human adipose-derived stem cells as a medicinal product toward wound healing

**DOI:** 10.1186/s13287-022-02928-7

**Published:** 2022-06-11

**Authors:** Guoqiang Ren, Qiuyue Peng, Trine Fink, Vladimir Zachar, Simone Riis Porsborg

**Affiliations:** Regenerative Medicine Group, Department of Health Science and Technology, Aalborg University, Fredrik Bajers Vej 3B, 9220 Aalborg, Denmark

**Keywords:** Potency assay, Wound healing, Chronic wounds, Adipose-derived stem cells, Stem cell-based medicinal product, Mode of action, Mechanism of action

## Abstract

In pre-clinical studies, human adipose-derived stem cells (hASCs) have shown great promise as a treatment modality for healing of cutaneous wounds. The advantages of hASCs are that they are relatively easy to obtain in large numbers from basic liposuctions, they maintain their characteristics after long-term in vitro culture, and they possess low immunogenicity, which enables the use of hASCs from random donors. It has been hypothesized that hASCs exert their wound healing properties by reducing inflammation, inducing angiogenesis, and promoting fibroblast and keratinocyte growth. Due to the inherent variability associated with the donor-dependent nature of ASC-based products, it appears necessary that the quality of the different products is prospectively certified using a set of most relevant potency assays. In this review, we present an overview of the available methodologies to assess the Mode and the Mechanism of Action of hASCs, specifically in the wound healing scenario. In conclusion, we propose a panel of potential potency assays to include in the future production of ASC-based medicinal products.

.

## Background

Disturbed wound healing is an element in the pathophysiology of a wide variety of medical conditions, such as anal fistulas, burn wounds, decubitus, and diabetic foot ulcers, jointly named chronic wounds. They are very burdensome for the patients and costly for society [1, 2]. Common for all of these chronic conditions is that the current treatments are ineffective and are accompanied by a general lack of evidence of an effect [3, 4]. New treatment strategies are widely sought, and some showing great potentials are stem cell-based approaches. Especially, human adipose-derived stem cells (hASCs) based treatment systems are of interest, as these stem cells have shown to be capable of repairing damaged tissue. This effect is due to their intrinsic ability to produce cytokines and other molecules necessary for wound healing and restoring the lost tissue functionality both in vitro, in vivo [5, 6], and in clinical trials (clinicaltrials.gov, NCT01932021, NCT02092870, NCT02099500, NCT02394873, NCT02314416). Furthermore, the high accessibility of hASCs, their relative ease of obtaining in large numbers from basic liposuctions, their considerable higher yield compared to other stem cell types, their ability to maintain their properties after long term in vitro culture, and their low immunogenicity makes them advantageous for large-scale production and regenerative therapy [6, 7].

When producing a stem-cell-based medicinal product, several considerations about the production phases and processes are to be taken [7–9]. Most importantly, the production must be in accordance with the current legislation by local authorities and the European Medicines Agency (EMA) or the U.S. Food and Drug Administration (FDA) [10, 11]. It is a legal requirement to evaluate the potency of each batch of a cellular therapy product, as these include an active substance for which the molecular structure cannot be fully defined. However, the legislation is flexible, as the adequacy of potency assays needs to be evaluated on a case-to-case basis. For a thorough review of the requirements and the experiences from the industry, please refer to the review by Bravery et al. [8]. In addition, when producing batches of a stem-cell-based medicinal product for clinical use, variability due to specific genetic profiles of individual donors or minor variations in the manufacturing process, such as different lots or types of reagents, may eventually introduce a non-negligible inconsistency [12].

Consequently, the properties of batches need to be formalized, and to this end, results from the selected most suitable potency assays should be included in the official batch release certification. Furthermore, the potency assays should give quantitative measures of the product characteristics and/or functions directly linked to the Modes (MoA) and/or Mechanisms (MeA) of Action of the medicinal product [13, 14]. In short, the MoA is the functional phenomenon induced by the ASCs as “increased angiogenesis,” and the MeA is the underlying molecular basis as “secretion of vascular endothelial growth factor (VEGF).”

In an attempt to facilitate the development of future potency assays within the area of chronic wounds, this review has critically gathered the current evidence regarding the hASC MoA and MeA and proposed a panel of potency assays to include in the production of future ASC-based medicinal products.

## Effect of ASCs in the different phases of wound healing

Typically, human wound healing is a rapid and very well-orchestrated multi-step process consisting of four consecutive phases: (i) hemostasis, (ii) inflammation, (iii) proliferation, and (iv) remodeling, which aim to restore skin integrity and thereby protect the organism from the external environment [15]. For a thorough review of the phases and the molecular events of wound healing, please see the review by Gonzalez et al*.* and Rodrigues et al*.* [16, 17]*.* The evidence gained from in vitro and in vivo testing indicates that hASCs can discontinue the otherwise prolonged inflammatory phase and stimulate the progression through the proliferative and remodeling phases [18–21].

### Inflammatory phase

During normal wound healing, the immune system is first activated to clear the wound for pathogens and cell debris and then switches to an anti-inflammatory state. In most chronic wounds, this progression stalls and leads to a continued pro-inflammatory environment. Hence, actively reducing the pro-inflammatory response and stimulating the transition toward an anti-inflammatory state is likely to accelerate the wound healing process. hASCs have been observed to modulate, especially monocytes, macrophages, and T cells, but also very sparse evidence shows an effect on natural killer cells, dendritic cells, and B-cells [21–24]. The hASCs induce this switch predominantly through the secretion of bioactive factors, but the effect of exosomes and direct cell–cell interaction has also been shown [18, 25–34]. These effects have been further corroborated by in vivo studies [25, 35, 36].

### Proliferative phase

During the proliferation phase, granulation tissue is formed, mainly by fibroblasts, and consists of extracellular matrix (ECM) containing, among others, proteoglycans, hyaluronic acid, collagen, and elastin [37]. Furthermore, the process of angiogenesis orchestrated by endothelial cells is crucial to restoring tissue vascularization after tissue damage. Likewise, to ensure the barrier function of the newly formed tissue and to reconstitute the protection of the underlying dermal structures, re-epithelialization achieved by activating the keratinocytes is essential [17].

For the role of hASCs in the proliferative phase, a plethora of studies have demonstrated that hASCs can be beneficial by enhancing the cellular processes related to the formation of granulation tissue, angiogenesis, and re-epithelialization [38, 39]. These effects are mediated both through the secretion of soluble factors such as cytokines and growth factors as well as through the deposition of structural proteins [32, 40].

### Remodeling phase

During the remodeling phase, reorganization, degradation, and synthesis of the ECM occur to ensure maximum tensile strength. Type III collagen undergoes degradation, and the synthesis of type I collagen is up-regulated. A reduction in the hyaluronic acid and fibronectin takes place through degradation by cells and matrix metalloproteinase (MMPs)[37]. Abnormal extracellular matrix deposition and remodeling will cause an undesired formation of hypertrophic scars or fibrosis, giving patients long-term complications [17]. To differentiate between the formation of granulation tissue and its remodeling using in vitro setups may not be straightforward, which is why the evidence of the effect of hASCs oftentimes does not discriminate between the phases. However, specifically for the remodeling phase, hASCs have exhibited the potential to affect the composition, deposition, and degradation of ECM, either through own production of ECM or modulation of ECM production by other cell types such as fibroblasts and keratinocytes [41, 42].

## Designing potency assays for wound healing

With the current understanding of the potential role of hASCs in wound healing, designing potency assays are the next step toward the development of an ASC-based therapy for chronic wounds.

Two types of potency assays can be implemented as part of the ASC product release protocol, the biological and the surrogate potency assays. The quantitative biological assays represent the traditional approach for assessing the potency of biological products. The purpose is to measure the activity of the product in relation to its MoA at a specific dose in a relevant biological system. Biological assays can include in vivo models, in vitro organ-, tissue-, or cell culture systems, or any combination of these [43].

When developing a suitable biological assay is not feasible, it may be necessary to identify a surrogate measurement of the biological activity. An advantage of a surrogate potency assay is a more straightforward experimental setup with lower variability [44]; however, it is essential that a correlation between the surrogate parameter and the biological activity related to potency is reliably established.

In many cases, a single biological or surrogate assay may not provide an adequate measure of potency. Thus, developing multiple complementary assays measuring different product attributes associated with quality, consistency, and stability may be necessary. Such a collection of assays, an assay matrix, might consist of a combination of biological assays, biological and surrogate assays, or surrogate assays alone [43].

### Biological potency assays

The in vivo models provide for the highest level of complexity and are closest to the actual clinical scenario. A number of in vivo trials have been conducted, using different animal models, testing the effect of hASCs on wound healing, and finding positive effects on all phases of this (Table 1). For the inflammation phase, decreased T cell infiltration and a decrease in the expression of pro-inflammatory genes as interferon-gamma (*INFG*) have been observed [25, 45]. For the proliferation phase, both an increase in collagen fibers, vascularity, and re-epithelialization have been detected [38, 46–49]. Across different models, the observed effect of ASCs on the remodeling phase was, in general, a reduced scar formation [38, 42, 46]. Yet, it is necessary to be aware of some non-negligible challenges associated with this approach. In particular, in the different animal models, varying modes of natural wound healing may take place, and ASCs may exert different MoAs in these. For example, it is well known that in rodents, wound contraction is the predominant mechanism of cutaneous healing, whereas in humans and other mammals, it is by the formation of granulation tissue and re-epithelialization [50]. Thereby mammal animal models may be considered more translatable in terms of the potency of hASCs in wound healing. Due to their labor-intensive nature, the in vivo assays are difficult to include in routine release testing. However, they are suitable for investigating the product efficacy, and they can therefore be included in the production pipeline as in-process controls to assess the potential effect of changes in manufacturing processes or materials [10].

**Table 1:** An overview of in vivo biological potency assays testing the effects of hASCs in animal models of wound healing

Phase	MoA	Animal model	References
*Inflammation*	↓ T cell infiltration, ↓ pro-inflammatory genes	SCID mice	[25]
	↓ T cell infiltration	Balb/C mice	[45]
*Proliferation*			
Granulation tissue formation	↑ collagenous fibers	Kunming mice	[46]
		Balb/c mice	[38]
Vascularization	↑ vascularity	Balb/c nude mice	[47]
		Kunming mice	[46]
		Diabetic mice	[48]
		CD1 athymic nude mice	[49]
Re-epithelialization	↑ re-epithelization	Kunming mice	[46]
		Diabetic mice	[48]
*Remodeling*	↓ scar formation	Kunming mice	[46]
	↑ collagenous fibers	Balb/c mice	[38]
	Flattened epidermis, ↑ bundles of collagen III, ↑ collagen III to I ratio, reticular collagen organization	Balb/c mice	[42]

hASCs, human adipose-derived stem cells; MoA, mode of action; ↑, increased; ↓, decreased

The in vitro potency assays directly measure the effect of the stem cell product on one or more target cells. Primarily used biological assays aim at quantifying the impact of the product on general cell characteristics, such as proliferation and migration. However, in the area of wound healing, it is also relevant to clarify the effect on wound healing phase-specific processes such as the activity of inflammatory cells, angiogenesis, and/or ECM production.

Currently, a multitude of different in vitro assays linked to the MoA has been published by laboratories worldwide. In the following, for cells relevant for each phase of wound healing, we will present a comprehensive overview of quantitative, relevant, and available biological targets and assays used to assess the MoA of hASCs published more than once.

To quantify the effect of hASCs on the inflammatory phase of wound healing, the most used assays are the 3H-thymidine or the carboxyfluorescein succinimidyl ester (CFSE) proliferation assays on peripheral blood mononuclear cells (PBMCs) or T cells (Table 2). As a measure of the macrophage polarization, the M1 to M2 ratio is often quantified by the level of proteins as arginase, the cluster of differentiation (CD) 206, interleukin (IL)-10, IL12, inducible nitric oxide synthase (iNOS), transforming growth factor-beta (TGF-β), and tumor necrosis factor-alpha (TNFα). The quantification of these is done using quantitative polymerase chain reaction (qPCR), enzyme-linked immunosorbent assay (ELISA), flow cytometry (FC), western blotting (WB), or immunofluorescence microscopy (IF).

**Table 2:** In vitro biological potency assays testing the effects of hASCs on cells relevant for the inflammation phase of wound healing

MoA	Target	Assay	References
PBMC proliferation	DNA	3H-Thymidine	[25, 51]
	Intracellular lysine residues	CFSE	[26]
T cell proliferation	DNA	3H-Thymidine	[27]
	Intracellular lysine residues	CFSE	[26, 28]
M1 to M2 transformation of macrophages	ARG1	qPCR	[31, 36]
	CD206	FC	[29–31]
		qPCR	[28]
	IL-10	ELISA	[32, 33]
		FC	[34]
		qPCR	[28, 31, 32]
		WB	[31]
	IL-12	IF	[31]
		qPCR	[34]
		WB	[31]
	iNOS	qPCR	[28, 31, 36]
	TGF-β	ELISA	[32, 33]
		qPCR	[28]
	TNFα	qPCR	[28, 29, 32, 34]
		ELISA	[29, 32, 33]

MoA, mode of action; PBMC, peripheral blood mononuclear cell; DNA, deoxyribonucleic acid; CFSE, carboxyfluorescein succinimidyl ester; ARG1, arginase 1; qPCR, quantitative polymerase chain reaction; CD, cluster of differentiation; FC, flow cytometry; IL10/12, interleukin 10/12; ELISA, enzyme-linked immunosorbent assay; WB, western blotting; iNOS, inducible nitric oxide synthase; TGF-β, transforming growth factor-beta; TNFα, tumor necrosis factor-alpha

To quantify the effect of hASCs on the proliferative phase, measures for both the granulation step, the angiogenesis, and the re-epithelialization are relevant (Table 3).

**Table 3:** In vitro biological potency assays testing the effects of hASCs in the proliferation phase of wound healing

MoA	Target	Assay	References
*Granulation tissue formation by fibroblasts*			
Proliferation	Dehydrogenases activity	CCK8	[46, 52–54]
	Cell number	Manual cell counting	[55–57]
	NAD(P)H-dependent dehydrogenase enzyme activity	MTT	[32, 45, 58, 59]
Viability			
Migration	Invasion	Scratch assay	[32, 38, 46, 53, 55, 56, 58, 60–63]
	Migration	Transwell assay	[38, 45, 46, 53]
ECM production	Collagen I	qPCR	[38, 39, 52, 55–57, 62, 63]
		WB	[52, 55, 57, 62]
		IF	[57]
	Collagen III	qPCR	[38, 52, 56, 57, 62]
		WB	[52, 57, 64]
		IF	[57]
	Elastin	qPCR	[38, 63]
		WB	[52]
	Fibronectin	qPCR	[62]
		WB	[52]
ECM modulation	MMP1	qPCR	[57, 62, 65]
		WB	[55]
	MMP2	qPCR	[52, 57, 65]
	TIMP1	ELISA	[65]
		qPCR	[57]
Paracrine milieu	VEGF	qPCR	[52, 63]
	TGF-β	qPCR	[52]
Proliferation		ELISA	[65]
*Vascularization by endothelial cells*			
Proliferation	NAD(P)H-dependent dehydrogenase enzyme activity	MTT	[61, 66]
	Dehydrogenases activity	CCK8	[46, 48, 52, 67]
Migration	Invasion	Transwell assay	[46, 48, 52, 67–69]
	Migration	Scratch assay	[46, 48, 52, 61, 66, 67, 70]
Angiogenesis	VEGFA	WB	[52]
		qPCR	[52, 70, 71]
	Sprouting	Aortic ring assay	[68, 72, 73]
	3D organization	Spheroid-based angiogenesis assay	[61, 74]
	2D organization	Tube formation assay	[46, 48, 52, 59, 66–69, 74–76]
*Re-epithelialization by keratinocytes*			
Proliferation	Dehydrogenases activity	CCK8	[52, 53, 77, 78]
	NAD(P)H-dependent dehydrogenase enzyme activity	MTS	[47, 79]
		MTT	[39, 45, 59]
Migration	MMP2	WB	[78]
		qPCR	[52]
	MMP9	WB	[78]
		qPCR	[52]
	Chemotaxis assay		[47, 80]
	Scratch assay		[47, 48, 53, 58, 61, 77–81]
	Transwell assay		[45, 52, 53, 77, 78]

MoA, mode of action; CCK8, cell counting kit-8; NAD(P)H, reduced nicotinamide adenine dinucleotide phosphate; MMT, 3-[4,5-dimethylthiazol-2-yl]-2,5 diphenyl tetrazolium bromide; qPCR, quantitative polymerase chain reaction; WB, western blotting; IF, immunofluorescence microscopy; MMP-1/2/9, matrix metalloproteinase-1/2/9; TIMP-1, TIMP metallopeptidase inhibitor 1; VEGFA, vascular endothelial growth factor A; TGF-β, transforming growth factor beta; 3/2 D, three/two dimensional, MTS, 3-(4,5-dimethylthiazol-2-yl)-5-(3-carboxymethoxyphenyl)-2-(4-sulfophenyl)-2H-tetrazolium

For the granulation step, the effect of hASCs on the proliferation of fibroblasts is mostly quantified using manual counting, the Cell Counting Kit-8 (CCK8) assay, and the 3-[4,5-dimethylthiazol-2-yl]-2,5 diphenyl tetrazolium bromide (MMT) assay. The latter is also used to quantify the effect on fibroblast viability. For assessing the effect on fibroblast migration, scratch and transwell assays are highly preferred. The formation of granulation tissue is heavily dependent on the production of ECM by fibroblasts. To evaluate the effect of hASCs on this, the ECM proteins, such as the collagen type I and III, elastin, and fibronectin are quantified by either qPCR or WB. Additionally, the modulation of ECM proteins may be examined through quantification of matrix metalloproteinase-1/2 (MMP-1/2) and TIMP metallopeptidase inhibitor 1 (TIMP1) by ELISA, qPCR, or WB. The effect of ASCs on the vascularization phase is primarily determined by means of endothelial cell proliferation in MTT or CCK8 assays, and migration assays, such as scratch and transwell assays. In addition, the level of VEGFA secreted by the endothelial cells is readily quantifiable by either qPCR or WB. Among more advanced approaches can be mentioned the highly popular tube formation assay, but also the aortic ring and the spheroid-based angiogenesis assays are amenable. The process of re-epithelialization is generally evaluated from the proliferation of the keratinocytes using the CCK8, 3-(4,5-dimethylthiazol-2-yl)-5-(3-carboxymethoxyphenyl)-2-(4-sulfophenyl)-2H-tetrazolium (MTS) or MTT assays. For epithelial migration as most suitable appear the chemotaxis, scratch, and transwell assays.

Regarding the remodeling phase, many of the assays used to evaluate the granulation step are also relevant for this phase (Table 4). However, it is important to recognize that the actual molecular processes taking place during these parts of wound healing are not identical. Some studies addressed this issue by electing a phase-specific fibroblast type as hypertrophic scar fibroblasts to better mimic in vivo situations (Table 5). The investigations are by large geared toward collagen type I and III depositions quantified by either qPCR, IF, or WB. Furthermore, the modulation of the ECM by MMP-1 and the scar contraction by smooth muscle α-actin (α-SMA) are mostly quantified using qPCR or WB.

**Table 4:** In vitro biological potency assays testing the effects of ASCs in the remodeling phase of wound healing

MoA	Target	Assay	References
Collagen deposition	Collagen I	qPCR	[38, 41, 52, 56, 57, 62–64]
		IF	[57]
		WB	[55, 57, 62, 64]
	Collagen III	qPCR	[38, 41, 52, 56, 57, 62, 64]
		IF	[57]
		WB	[57, 64]
ECM modulation	MMP-1	qPCR	[57, 62, 65]
		WB	[55]
Scar contraction	α-SMA	qPCR	[56, 64]
		WB	[41, 64]

MoA, mode of action; qPCR, quantitative polymerase chain reaction; IF, immunofluorescence microscopy; WB, western blotting; MMP-1, matrix metalloproteinase-1; α-SMA, Smooth muscle α-actin

**Table 5:** Cell types used in the in vitro assays testing the effects of ASCs in the different phases of wound healing

Cell type	Cell model	References
Macrophage	Primary human macrophage	[31, 33]
	Primary mice macrophage	[28–30, 36]
	RAW 264.7 macrophages	[33]
Fibroblast	Human dermal fibroblasts	[38, 39, 45, 46, 52, 53, 55, 57, 58, 60–65]
	Mouse skin fibroblasts	[56]
	MSU-1.1 fibroblasts	[59]
	L929 fibroblasts	[32]
	Hypertrophic scar fibroblasts	[41]
Endothelial cell	HDMEC	[46, 69, 70]
	HUVEC	[48, 52, 61, 67, 68, 73–76, 83]
	Primary rat endothelial cell	[47]
	Ea.hy926	[66]
	HskMEC.2	[59]
Keratinocyte	Primary human keratinocyte	[47, 61, 80, 81]
	HaCAT	[45, 48, 52, 53, 58, 77–79]

HDMEC, human dermal microvascular endothelial cells; HUVEC, human umbilical vein endothelial cells; HskMEC.2, human Skin Microvascular Endothelial Cells.2

One of the crucial elements of a potency assay is the target cell, which accounts for the biorelevant test system. Identifying the target cell to be used to measure the effect of the stem cell product in in vitro potency assays is essential, and considerations like availability, cell characteristics, growth, morphology, and assay compatibility.

In the assays suggested above, a variety of target cells has been used, and their relevance and fit must be evaluated for each assay and each clinical case (Table 5).

As a model for macrophages, both primary human and mouse macrophages have been used. Additionally, the RAW 264.7 macrophages have been used, which are monocyte/macrophage-like cells originating from Abelson leukemia virus-transformed cell line derived from BALB/c mice [82].

To assess the effect on fibroblasts, fibroblasts of different origins have been used, including human dermal fibroblasts, the human fibroblast cell strain MSU-1.1, mouse skin fibroblasts, the mouse fibroblasts cell line L929, and hypertrophic scar fibroblasts.

The endothelial cells used include both primary endothelial cells as Human Dermal Microvascular Endothelial Cells (HDMEC), Human Umbilical Vein Endothelial Cells (HUVEC), rat endothelial cells, and immortalized endothelial cell lines as the immortalized human umbilical vein endothelial cell line Ea.hy926, and the human immortalized skin microvascular endothelial cells HskMEC.2.

Finally, different types of keratinocytes or models thereof were used to investigate the effect of ASCs. That being Primary human keratinocytes or the spontaneously transformed aneuploid immortal keratinocyte cell line derived from adult human skin HaCAT.

### Surrogate potency assays

A surrogate for potency assay measures a parameter known to correlate with a functional effect. Typical for all surrogate assays is that the measurement is not made on a target cell but on the medicinal product itself, in this case, the hASCs. The surrogates can essentially be divided into three categories: surface markers, gene transcription, and soluble mediators.

#### Surface markers

For the detection of hASC surface markers, FC appears to be the preferred technique. It has been routinely used in the quality control of the identity and purity of ASCs according to the criteria established by the International Federation for Adipose Therapeutics and Science (IFATS) and the International Society for Cell and Gene Therapy (ISCT) [84]. Recently, a great deal of effort has been directed toward uncovering the functional role of an ever-increasing number of cell surface markers, and fluorescence-activated cell sorting (FACS) is the method of choice to explore specific cell subpopulations [85–88].

In terms of angiogenesis, especially the surface marker CD248 has been given a lot of attention, as the use of hASCs expressing this marker has been found to entail a much higher presence of vascularity in wounds [49]. Additionally, hASCs expressing the surface marker SSEA-4 have been shown to hold a high endothelial differentiation capacity [89]**,** and the CD34^+^CD31^−^ hASC subpopulation has been found to significantly improve blood flow and neovascularization [90]. Furthermore, a specific complex phenotype, characterized by a CD73^+^CD90^+^CD105^+^CD34^−^CD146^+^CD271^−^ combination, has been confirmed superior in stimulating endothelial cell migration [88].

Relevant for the granulation tissue formation by fibroblasts the ASC subpopulation characterized as CD274^+^ CD146^+^ was found superior in stimulating fibroblast migration [87]**.**

Despite clear progress in this field, the evidence that given surface markers are linked to defined functions needs further scrutiny. There are legitimate concerns regarding the standardization of the lineage specifications since there are variations in cytometer components and experimental setups across laboratories and the general cross-bleed compensation issues. Especially when it comes to the detection of low-expressed markers, the delineation of the positive boundary of the targeted markers may become burdened by a notable error. Lastly, it should be reiterated that it is the end-product that is to be tested, as the phenotype of hASCs evolves during expansion and cell manipulation [88].

#### Gene transcription

To evaluate the potency of ASCs by assessing the expression of individual genes, qPCR has been widely used. However, the microarray technique can provide an edge if parallel analysis of a large number of genes in very few cells is intended [91, 92]. Yet, more advanced is RNA sequencing, which enables comprehensive quantification of the whole transcriptome within a wide dynamic range.

As surrogates for the effect of hASCs on the inflammatory phase, the expression of the C-X-C motif chemokine 10 (*CXCL10*), *TGFβ*, and *IDO* genes could be examined [25]. The *IDO* has already been verified to play an essential role in the hASC-mediated immunosuppression [93]. Other cytokine genes involved in the regulation of inflammatory responses, including *TNF, IL6, IL4,* and *IL10,* also appear to be suitable candidates [32]. For the proliferative phase, the transcriptional activation of the *MALAT1* gene has been verified to be linked to the hASC potential to enhance human dermal fibroblast migration [60]. Furthermore, the pro-angiogenic genes Angiopoietin-1 (*Ang1*)*,* Insulin-like growth factor 1 (*IGF1*)*,* or *VEGFA* have been shown to be associated with the angiogenic properties of hASCs [72, 76]. For the remodeling phase, no correlations between the gene expression and function have been identified.

Overall, there is an opacity of evidence linking gene regulation with the wound healing functions of hASC. The difficulty in identifying valid correlations may reside in the fact, that the transcriptional activation is not always proportionally reflected in the protein-based functionality due to various post-transcriptional and post-translational effects.

#### Soluble mediators

To evaluate the hASC wound healing potency based on their secretome, various methods are available. The antibody-based techniques offer definite advantages in their high sensitivity, specificity, reproducibility, and broad dynamic range. Consequently, a single analyte can readily be quantified using ELISA or WB [94]. The non-antibody-based approaches, such as the mass spectrometry or liquid chromatography-mass spectrometry (LC–MS/MS), make possible, on the other hand, a comprehensive analysis of complex protein mixtures, which may be useful if the discovery of unknown mediators or pathways is the primary goal. Using these tools thousands of proteins have been found secreted by ASCs [40, 95, 96].

Of the soluble factors secreted by hASCs relevant to the inflammatory phase, indoleamine 2,3-dioxygenase (IDO) appears of significance since it has been reported to mediate the hASC immunomodulatory effect and be responsible for the suppression of PBMC proliferation and T cell infiltration [93, 97, 98]. In the proliferative phase, the VEGF may be of importance since it has a permissive effect on the angiogenic response [72]**,** and galectin-1 has shown to carry out a stimulating effect on the migration of keratinocytes [58]. Finally, in the remodeling phase, no factors have been verified in the perspective of wound healing, but prolyl 4-hydroxylases 1 and 2 (P4HA1 and 2), procollagen-lysine 2-oxoglutarate 5-dioxygenase 1 (PLOD1), and alpha-1 chains of collagen types I, III, and VII may be involved since they have the capacity to regulate the ECM [96]. Similarly, cathepsin L and MMP1 may play a role in the regulation of the ECM [99].

**Fig. 1:**
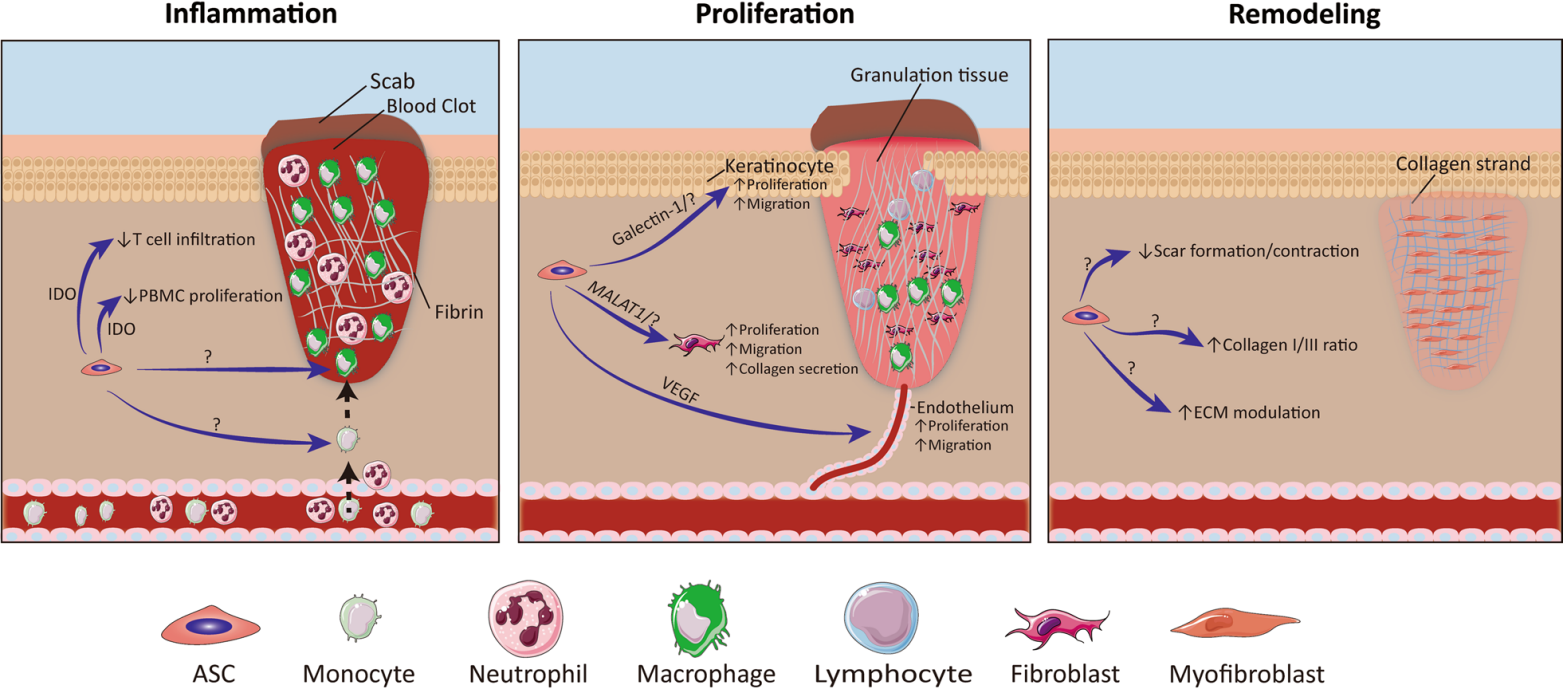
Confirmed MeA of adipose-derived stem cells in the different phases of wound healing. Adipose-derived stem cells (ASCs) have distinct mode of actions (MoA) in each stage of wound healing, however the mechanism of action (MeA) by which they execute these are still not fully validated. In the Inflammation stage it has been confirmed that ASCs secrete IDO, and through that having an immunosuppressive effect on T cell infiltration and PBMC proliferation. In the proliferation stages, ASCs are through galectin-1, *MALAT1,* and VEGF confirmed to increase re-epithelialization, ECM regulation, and angiogenesis, respectively. However, in the remodeling stage no factors have been verified for the different MoAs, including reducing scar formation, reducing scar contraction, increasing the ratio of type I/III collagen, and a general ECM modulation. IDO, indoleamine 2,3-dioxygenase; PBMC, peripheral blood mononuclear cell; MALAT1, mucosa-associated lymphoid tissue lymphoma translocation protein 1; VEGF, vascular endothelial growth factor; ECM, extracellular matrix

In summary, many indications of mechanisms of action of hASCs have been put forward; however, only a few of these have, across all three categories of surrogate potency assays, been correctly verified (Table 6). For surrogate for potency assays, it is required to demonstrate a direct link with the intended biological activity and the MoA. This link is established through knockout or signaling pathway inhibition assays to ensure that the molecule of interest is indeed responsible for the effect observed. When putting these verified factors in perspective of all the identified MoA of ASCs in wound healing, it becomes apparent that a lot of research and validation is still missing before a direct link between the MoAs and MeAs can be made. Especially for the remodeling phase, the evidence is very deficient (Fig. 1).

**Table 6:** Confirmed mechanisms of action of hASCs

MoA	MeA	Confirmed by	References
Proliferation of PBMCs	Secretion of IDO	IDO activity inhibitor L-tryptophan	[93]
		IDO activity inhibitor Kynurenine	[93]
		IDO activity inhibitor epacadostat	[98]
Infiltration of T cells	Secretion of IDO	IDO activity inhibitor L-tryptophan	[97]
Migration of fibroblasts	Expression of *MALAT1*	ASO transfection	[60]
Endothelial sprouting	Secretion of VEGF	VEGFR inhibitor SU5416	[72]
Migration of keratinocytes	Secretion of galectin-1	Neutralizing antibody	[58]

MoA, mode of action; MeA, mechanism of action; PBMS, Peripheral blood mononuclear cells; IDO, indoleamine 2,3-dioxygenase; MALAT1, mucosa-associated lymphoid tissue lymphoma translocation protein 1; ASO, antisense oligonucleotides; VEGF, vascular endothelial growth factor

## Conclusion

A plethora of pre-clinical and clinical studies laid the foundation for hASCs to be hailed as the future cellular therapy product for healing chronic wounds. As a cell-based medicinal product, different batches prior to release for clinical use need to be found to conform to the quality requirements, including, among others, potency tests to verify the stability and consistency of the product. Based on the recommendations by the ISCT, an individual assay is unlikely to adequately cover the product attributes predicting the clinical utility [12]. Consequently, the ultimate panel should be in the form of a matrix of several potency assays capturing the critical activities of the product MeA and MoA. At this date, the biological assays appear to be firmly established; however, more work is needed to confirm the role of single surrogate factors and their correlation with the overall effect. Lastly, when electing for the in vitro assay panel, it cannot be overemphasized that an appropriate cell type is chosen since the primary cells clearly surpass the cell lines in mimicking the in vivo processes.

## Data Availability

Not applicable.
